# Supplementary material for: Identification of photoperiod-induced specific miRNAs in the adrenal glands of Sunite sheep (Ovis aries)
Source: Front Vet Sci. 2022 Jul 22;9:888207. doi: 10.3389/fvets.2022.888207 (PMC9354845; doi:10.3389/fvets.2022.888207)
Supplement: Supplementary file 1 [file Data_Sheet_1.ZIP › Supplementary file/miRDeep2 file.zip!/miRDeep2 file/Know_Structure/SP42A3/oar-let-7b.pdf]

miRBase precursor : oar-let-7b
Total read count : 204872
oar-let-7b read count : 204074
remaining reads : 798

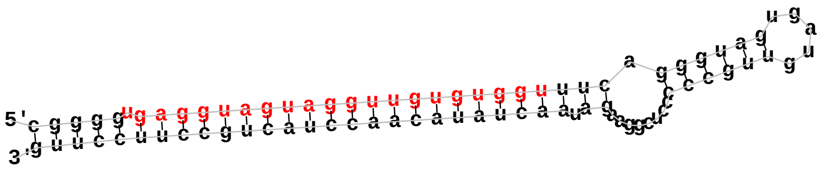

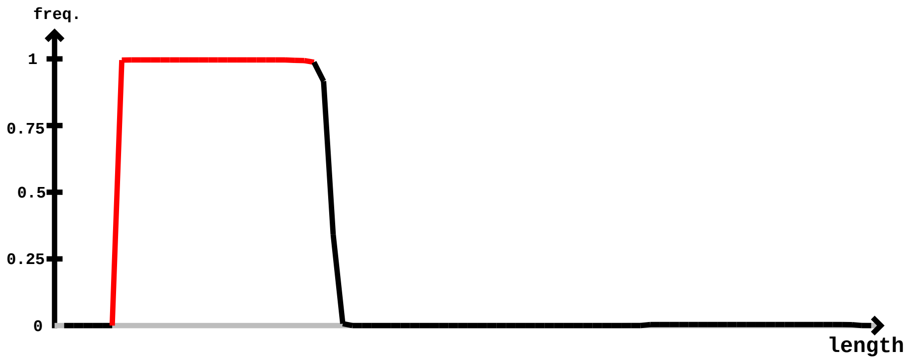


```
      oar-let-7b
5'- cggggugagguaguagguugugugguuucaggguagugauguugcccccucggaagauaacuauacaaccuacugccuuccuug   -3'    exp
    (((((.(((((((((((((((((((((((.((((((.....))))))(....)..)).))))))))))))))))))))))))))    reads      mm        sample
    ....gugagguaguagguugugugguu.........................................................    1          0         seq
    ....gugagguaguagguugugugguuu........................................................    1          0         seq
    .....ugaCguaguagguugug..............................................................    1          1         seq
    .....ugagguaguagguugug..............................................................    23         0         seq
    .....ugagguaguagguugugu.............................................................    305        0         seq
    .....ugagguaguaAguugugu.............................................................    1          1         seq
    .....ugagguaguagguugugug............................................................    184        0         seq
    .....ugagguaguagguugugugg...........................................................    1255       0         seq
    .....ugagguaguagguuguguggu..........................................................    14559      0         seq
    .....ugagguaguagguugugugguu.........................................................    117676     0         seq
    .....ugagguaguagguugugugguuu........................................................    68493      0         seq
    .....ugagguaguagguugugugguuuc.......................................................    1434       0         seq
    .....ugagguaguagguugugugguuuca......................................................    7          0         seq
    ......gagguaguagguuguguggu..........................................................    9          0         seq
    ......gagguaguagguugugugguu.........................................................    77         0         seq
    ......gagguaguagguugugugguuu........................................................    46         0         seq
    ......gagguaguagguugugugguuuc.......................................................    1          0         seq
    .......agguaguagguugugugguuuc.......................................................    1          0         seq
    ...........................ucaggguagugauguugcccccuc.................................    1          0         seq
    ...........................ucaggguagugauguugcccccucggaagau..........................    1          0         seq
    ............................................cccccucggaagauaacuauac..................    1          0         seq
    ..............................................cccucggaagauaacuauacaacc..............    2          0         seq
    ...............................................ccucggaagauaacuauaca.................    1          0         seq
    ......................................................agauaacuauacaaccuacugccu......    1          0         seq
    ........................................................auaacuauacaaccuacugccuuc....    1          0         seq
    .........................................................uaacuauacaaccuacugccuuc....    2          0         seq
    .........................................................uaacuauacaaccuacugccuucc...    1          0         seq
    ..........................................................aacuauacaaccuacugccuuc....    1          0         seq
    ..........................................................aacuauacaaccuacugccuucc...    2          0         seq
    ............................................................cuauacaaccuacugccuuc....    8          0         seq
    ............................................................cuauacaaccuacugccuucc...    106        0         seq
    ............................................................cuauacaaccuacugccuuccu..    630        0         seq
    ............................................................cuauacaaccuacugccuuccuu.    31         0         seq
    ............................................................cuauacaaccuacugccuuccuug    1          0         seq
    .............................................................uauacaaccuacugccuucc...    4          0         seq
```

oar-let-7b

```
cggggugagguaguagguuguguggguuucaggguagugauguugcccccucggaagauaacuauacaaccuacugccuuccuug
.............................................................uauacaaccuacugccuuccu..    3          0          seq
..............................................................auacaaccuacugccuuccu..    1          0          seq
```
